# Readers as research detectives

**DOI:** 10.1186/1745-6215-10-2

**Published:** 2009-01-07

**Authors:** Peter C Gøtzsche

**Affiliations:** 1 The Nordic Cochrane Centre, Rigshospitalet, Dept 3343, Blegdamsvej 9, DK-2100, Copenhagen Ø, Denmark

## Abstract

Flaws in research papers are common but it may require arduous detective work to unravel them. Checklists are helpful, but many inconsistencies will only be revealed through repeated cross-checks of every little detail, just like in a crime case. As a major deterrent for dishonesty, raw data from all trials should be posted on a public website. This would also make it much easier to detect errors and flaws in publications, and it would allow many research projects to be performed without collecting new data. The prevailing culture of secrecy and ownership to data is not in the best interests of patients.

## Background

We build on the scientific results of others when we choose treatments and plan new research. It is a challenge, however, how to do this most effectively and reliably, as flaws in research are common.

Most users of the scientific literature read vastly more conclusions than they read abstracts, and vastly more abstracts than full papers. This approach is difficult to avoid, but it is a high-risk strategy. Although our intention might be to use conclusions and abstracts only as screening tests, helping us to identify those papers that merit a closer look, it is impossible to actively suppress what we have just "learnt" from a cursory reading. The human brain doesn't work that way, in fact, it is remarkably good at memorizing things we have been exposed to in a flash.

Conclusions are the worst part of papers, and they are often tainted by wishful thinking, academic ambition, or the influence of money. Commercial pressures may explain, for example, why conclusions in randomised trials recommended the experimental drug as the drug of choice much more often if the trial was funded by for-profit organisations, even after adjustment for the effect size (odds ratio 5.3) [1].

Abstracts may not be any better. A review of 520 randomly selected abstracts found that the first result in the abstract was statistically significant in 70% of the trials, 84% of cohort studies, and 84% of case-control studies [2]. Although many of these results were derived from subgroup or secondary analyses, or biased selection of results, they were presented without reservations in 98% of the trials. As true progress in therapeutics or in scientific knowledge is relatively rare, these results indicate that significant P-values in abstracts should generally be disbelieved.

Because of the many problems in research papers, the task of reviewing research critically is important and has developed into a science of its own. But it took time to gain momentum. Gene Glass coined the term *meta-analysis *in 1976 [3], but even as late as in 1990, one would find only about 150 meta-analyses on MEDLINE. Most of the essential knowledge we now have about bias in research papers comes from methodological studies published within the last 15 years. This knowledge has mainly been derived from empirical studies of randomised trials but it is also useful when evaluating results of observational research.

The methodological studies have made it clear that a failed study can be so nicely dressed up that most readers would think the study is reliable, somewhat like a *lit de parade *where one might think that the deceased, lying peacefully in his or her best clothes, is not dead, but only sleeping.

The deeper one digs, the more one finds. Authors of systematic reviews and other critical readers of scientific papers may therefore wish to take on the role as research detective, which has similarities to the work of a police detective. First of all, no matter how respected the authors, their institution and the journal, the starting point should be that important evidence might have been covered up, and that people could be lying. We once asked 102 trialists whether they had measured outcomes they had not reported. Only 48% responded, and 86% of the remaining authors initially denied the existence of unreported outcomes, prior to receiving our list of unreported outcomes that we had compiled from the trial protocols [4]. We do not interpret this as lying but rather as an example that people tend to reply to questionnaires in a way that shows themselves in a favourable light, in particular when they think it would not be possible to check their replies.

Second, it is hard to find out what is *not *there, but which should have been there, whether it is a nicely cleaned research paper or a nicely cleaned crime site. Checklists are helpful, but mostly for inexperienced detectives. Quite often, something is wrong that will not be captured by a checklist, but which is more likely to be revealed the more experience the detective has, the more time he devotes to the case, and the more pedantic he is, checking every little detail again and again, also against other details, as there is an important learning process that necessitates going back to issues one has already dealt with.

## Discussion

The case study recently published in *Trials *by Karim Hirji [5] is an example that many important problems can sometimes be found with a trial if one is persistent. What makes the case particularly interesting are that most of the problems would not have been captured by a checklist, and that both participants at a course on evidence-based medicine (who are usually very good at spotting problems together) and authors of systematic reviews consistently considered the trial to be of high quality, although it might have been fatally flawed in several respects.

Several of the flaws were well hidden and Hirji used three months on his investigations, which would not be practicable or cost-effective if this were to be the norm. But we can still do a good job if we share the workload by co-operating, like researchers do in The Cochrane Collaboration, where the general idea is that there should be only one systematic review on a particular issue, which gets updated if readers alert the authors to important problems they have missed.

We may hope that Hirji's case was atypical, but I doubt it. Many of the issues Hirji uncovered are common in trial reports, e.g. lack of information on exactly which data were missing when, non-adherence to the trial protocol, erroneous coding of events, misleading statistical analyses and a general lack of understanding of basic statistical principles. A thesis based on a systematic review of 196 head-to-head trials of non-steroidal, anti-inflammatory drugs revealed an overwhelming amount of bias, much of which was covert and only detected because results of meta-analyses agreed poorly with what was claimed in the individual trial reports [6].

*Trials *contributes to correcting the scientific record by welcoming submissions that discuss trials published elsewhere. This is important, as it is usually impossible to get relevant criticism accepted in the journal that published the trial after the time limit for publishing letters has passed, which it does in only two weeks for some journals, although oversees subscribers may still not have received the journal. It is difficult to see any good rationale for time limits for comments that demonstrate fatal flaws. Indeed, it is similar to dismissing a police detective's proof of the identity of the killer because a judge or a jury had opined that the killer was not-guilty, based on existing evidence. Scientific cases should have no closure date.

Important progress has been made recently towards greater transparency and less bias in trials. Many journals refer to the CONSORT statement in their instructions to authors that aim at improving clarity in reporting [7], the BMJ [8] and a few other journals now require trial protocols for submission of trial reports, JAMA will not publish an industry-sponsored trial unless the data analysis was conducted by an independent statistician at an academic institution [9], and several journals will not publish a trial unless it was registered at inception [10]. Furthermore, work is ongoing aiming at ensuring free access to trial protocols and to all results from all trials.

## Conclusion

I agree with Hirji's suggestion that raw data from all trials should be posted on a public website. This would be a major deterrent for flawed analyses and reports, and it would be much easier to detect errors and flaws in publications. It would also be tremendously cost-effective, as many relevant research projects could be performed by using data that have already been collected for another purpose. It is time to change the prevailing culture of secrecy and ownership to data, as this is clearly not in the best interests of the patients.

## Competing interests

The author declares that they have no competing interests.
